# Significant impact of miRNA–target gene networks on genetics of human complex traits

**DOI:** 10.1038/srep22223

**Published:** 2016-03-01

**Authors:** Yukinori Okada, Tomoki Muramatsu, Naomasa Suita, Masahiro Kanai, Eiryo Kawakami, Valentina Iotchkova, Nicole Soranzo, Johji Inazawa, Toshihiro Tanaka

**Affiliations:** 1 Department of Human Genetics and Disease Diversity, Graduate School of Medical and Dental Sciences, Tokyo Medical and Dental University, Tokyo 113-8510, Japan; 2 Laboratory for Statistical Analysis, RIKEN Center for Integrative Medical Sciences, Yokohama 230-0045, Japan; 4 Department of Molecular Cytogenetics, Medical Research Institute and Graduate School of Medical and Dental Science, Tokyo Medical and Dental University, Tokyo 113-8510, Japan; 5 Advanced Medicinal Research Laboratories, Tsukuba Research Institute, Ono Pharmaceutical CO., LTD., Tsukuba 300-4247, Japan; 6 Laboratory for Disease Systems Modeling, RIKEN Center for Integrative Medical Sciences, Yokohama 230-0045, Japan; 7 Human Genetics, Wellcome Trust Sanger Institute, Genome Campus, Hinxton, CB10 1HH, UK; 8 Department of Haematology, University of Cambridge, Hills Rd, Cambridge CB2 0AH, UK; 9 Bioresource Research Center, Tokyo Medical and Dental University, Tokyo 113-8510, Japan; 10 Laboratory for Cardiovascular Diseases, RIKEN Center for Integrative Medical Sciences, Yokohama 230-0045, Japan

## Abstract

The impact of microRNA (miRNA) on the genetics of human complex traits, especially in the context of miRNA-target gene networks, has not been fully assessed. Here, we developed a novel analytical method, MIGWAS, to comprehensively evaluate enrichment of genome-wide association study (GWAS) signals in miRNA–target gene networks. We applied the method to the GWAS results of the 18 human complex traits from >1.75 million subjects, and identified significant enrichment in rheumatoid arthritis (RA), kidney function, and adult height (*P *< 0.05/18* *= 0.0028, most significant enrichment in RA with *P *= 1.7 × 10^−4^). Interestingly, these results were consistent with current literature-based knowledge of the traits on miRNA obtained through the NCBI PubMed database search (adjusted *P *= 0.024). Our method provided a list of miRNA and target gene pairs with excess genetic association signals, part of which included drug target genes. We identified a miRNA (miR-4728-5p) that downregulates *PADI2*, a novel RA risk gene considered as a promising therapeutic target (rs761426, adjusted *P *= 2.3 × 10^−9^). Our study indicated the significant impact of miRNA–target gene networks on the genetics of human complex traits, and provided resources which should contribute to drug discovery and nucleic acid medicine.

MicroRNA (miRNA), a small non-coding RNA molecule of approximately 22 nucleotides, regulates degradation and translational repression of a specific gene through its binding to the 3′ UTR of target mRNA.^1^ MiRNA has essential impacts on the pathogenesis of human complex traits, including cancers, cardiovascular diseases, and autoimmune diseases; thus, can act as a disease biomarker as well as a therapeutic target^1^,^2^. To date, approximately 2,000 human miRNAs have been annotated in the miRNA registry (miRBase), targeting and regulating majority of the coding genes^3^. Recent technological development has enabled the identification of additional functional miRNA^4^, thereby increasing the impact of miRNA in the field of bioscience.

The regulatory effect of miRNA is a heritable genetic trait^5^. Previous studies investigated the contributions of human genetic polymorphisms to miRNA functions, by surveying single nucleotide polymorphisms (SNPs) that alter miRNA seed or target sites^6^ or by conducting expression quantitative trait (eQTL) analyses of miRNAs^7^. These approaches have identified several empirical examples that could link SNPs to human disorders; for example, a synonymous variant in *IRGM* confers a risk for Crohn’s disease by altering a miR-196 binding site^8^. However, in comparison with the progress achieved in the field of mRNA epigenomics, the comprehensive landscape regarding the impact of miRNA on genetics of human complex traits has not been fully elucidated.

A challenge in miRNA epigenomics is the complexity of miRNA–target gene networks. Given the vast amount of potential combinations of miRNAs and target genes, systematic computational predictions of miRNA–target genes are necessary. However, current target gene prediction algorithms include uncertainty in their accuracy, which is represented by the output of quantitative prediction scores that are inconsistent among algorithms^9^. Integration of this high-dimensional network information with existing genetic or other epigenetic resources will require novel bioinformatics approaches.

Here, we report a novel analytical method to comprehensively evaluate the enrichment of genome-wide association study (GWAS) signals in miRNA–target gene networks (miRNA–target gene enrichment analysis in GWAS; **MIGWAS**). The application of our method in large-scale GWAS results of human complex traits could provide an empirical and quantitative estimation of the impact of miRNA–target gene networks on the genetics of these human complex traits. Our method also provides a list of miRNA and target gene pairs with excess genetic association signals, which may contribute to the discovery of therapeutic miRNAs and drug target genes.

## Results

### Summary of the MIGWAS analytical method

The principal hypothesis of our method was that, for human complex traits in which miRNA plays important biological roles, the association signals observed in large-scale GWASs would be relatively enriched for miRNA and target gene pairs. To this end, we constructed an *in silico* pipeline (MIGWAS) to systematically evaluate whether the trait association signals of miRNA and target gene pairs were more likely to both demonstrate significant associations than expected by chance. Considering that the top-associated SNPs identified in the GWAS studies can only partially explain genetic heritability, we utilized genome-wide SNP p-values obtained in the GWAS to annotate miRNA- and gene-based association signals (= *P*_miRNA_ and *P*_gene_, respectively)^10^. To account for the uncertainty of miRNA–target gene predictions, we integrated the analytical results by sequentially sliding the prediction score thresholds obtained from multiple prediction algorithms (miRDB^11^, MiRmap^12^, PITA^13^, and TargetScan^14^; Supplementary Table 2). Quantitative estimates of fold changes in association signal enrichment (= *F*_enrichment_) and their significance (= *P*_enrichment_) were evaluated using a permutation procedure. The source codes for the MIGWAS method and the data resources are available upon request to the authors.

### No enrichment was observed in the null GWAS data

We first confirmed that our method did not report spurious enrichment results, even in the condition that strong inflation exists in the original GWAS result due to reasons such as population stratification. As a negative control of our method, we generated null GWAS results using 1000 Genomes Project Phase I (α) European genotype data. When we applied our method to the null GWAS results, we did not observe a significant enrichment of the association signals in the miRNA and target gene pairs (*F*_enrichment_* *= 0.55~1.42 and *P*_enrichment_* *= 0.012~0.89; *n *= 5; Supplementary Figure 1A). Similarly, when we artificially induced inflation of the GWAS association signals, by inversely applying genomic control (GC) corrections with λ_GC_ values in the range of 1.0–3.0, we did not observe significant enrichment (*F*_enrichment_* *< 0.99 and *P*_enrichment_ > 0.25; Supplementary Figure 1B). These results empirically demonstrated the statistical robustness of our method.

### Significant impact of miRNA–target gene networks on human complex trait genetics

We then applied our MIGWAS method to previously published large-scale GWAS results of human complex traits. We collected GWAS results of 18 human complex traits that comprised a total of > 1.75 million individuals. These traits included anthropometric traits,^15^,^16^ hematological parameters,^17^,^18^ biochemical parameters,^19^,^20^ physiological functions,^21^,^22^ metabolic diseases,^23^,^24^ psychiatric diseases,^25^,^26^ immune-related diseases,^27^,^28^ and others^29^ (Supplementary Table 1).

**Figure 1:**
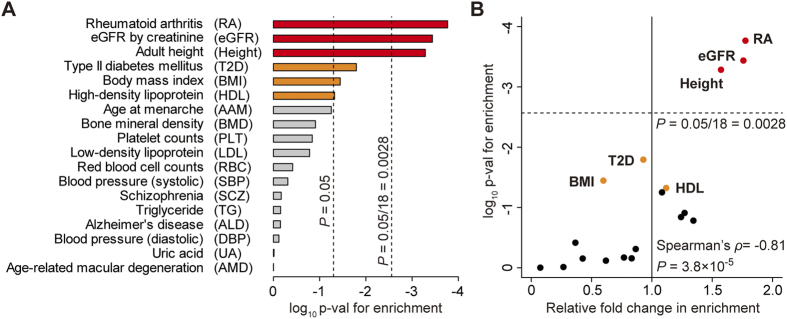
GWAS signal enrichment in miRNA–target gene networks. Significance (= *P*_enrichment_; **A**) and relative fold changes (= *F*_enrichment_; **B**) in the enrichment of GWAS association signals of each human complex trait on miRNA–target gene networks. *P*_enrichment_ and *F*_enrichment_ significantly correlated (*P *= 3.8 × 10^−5^). Rheumatoid arthritis (RA), estimated glomerular filtration rate (eGFR), and adult height exhibited significant enrichment (*P*_enrichment_* *< 0.05/18* *= 0.0028) with relative fold changes greater than 1.5-fold when compared to the null hypothesis (colored red).

**Figure 2:**
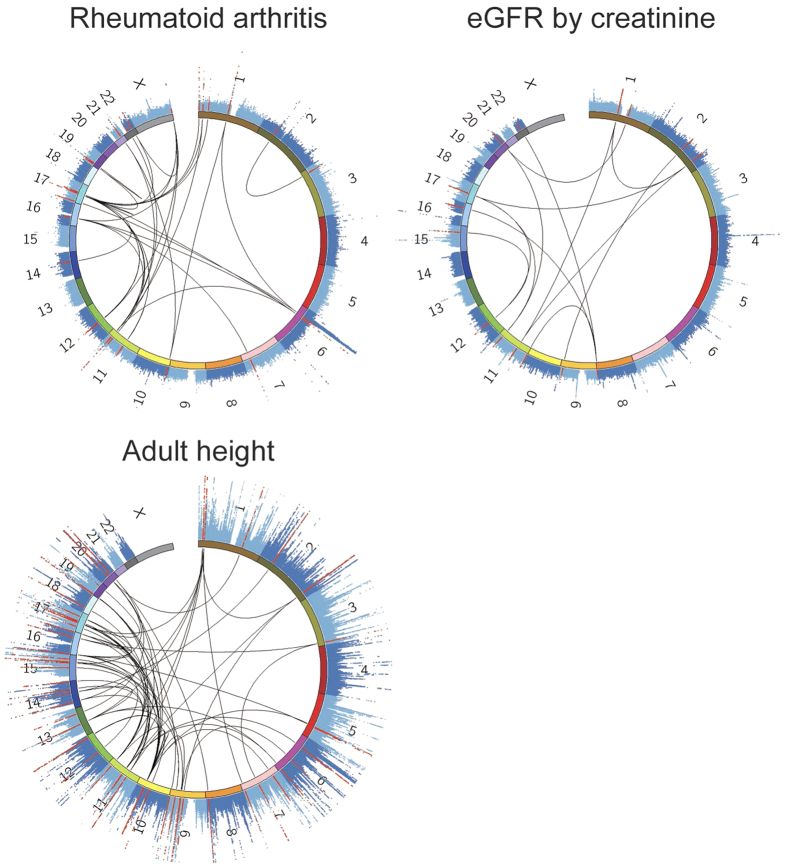
miRNA–target gene networks in the GWAS of human complex traits. CIRCUS plots^45^ of the GWAS association signals and miRNA–target gene networks. Manhattan plots representing the GWAS results are indicated as the outer layers of the CIRCUS plots^45^. MiRNA and target gene pairs for which both the *P*_miRNA_ and *P*_gene_ satisfied the nominal association threshold (α* *= 0.01) and for which the prediction scores were within the top 1st percentile in multiple prediction algorithms are connected by lines. SNPs located within ±150 kbp of these miRNAs or genes are colored red in the Manhattan plots.

Of the 18 examined human complex traits, rheumatoid arthritis (RA), estimated glomerular filtration rate (eGFR), and adult height exhibited significant enrichment of the association signals in the miRNA and target gene pairs (*P*_enrichment_* *< 0.05/18* *= 0.0028; Fig. 1A). The significance and fold changes of this enrichment correlated significantly among the traits (Spearman’s *ρ *= −0.81; *P *= 3.8 × 10^−5^; Fig. 1B). For the top three traits (RA, eGFR, and adult height), the relative enrichment was more than 1.5-fold higher in the miRNA–target gene association signals in the GWAS when compared to the null hypothesis (*F*_enrichment_ > 1.57). In particular, the most significant enrichment was observed in RA (*F*_enrichment_* *= 1.77, *P*_enrichment_* *= 1.7 × 10^−4^). Suggestive enrichment was observed for the metabolic traits, including type II diabetes mellitus (T2D), body mass index (BMI), and high-density lipoprotein (HDL; *P*_enrichment_* *< 0.05). On the other hand, the least enrichment was observed for age-related macular degeneration (AMD) and uric acid (UA; *P*_enrichment_ > 0.97). These results suggest a significant impact of miRNA–target gene networks on the genetics of a variety of human complex traits (Fig. 2).

### The MIGWAS result was supported by literature-based knowledge on the traits

**Figure 3:**
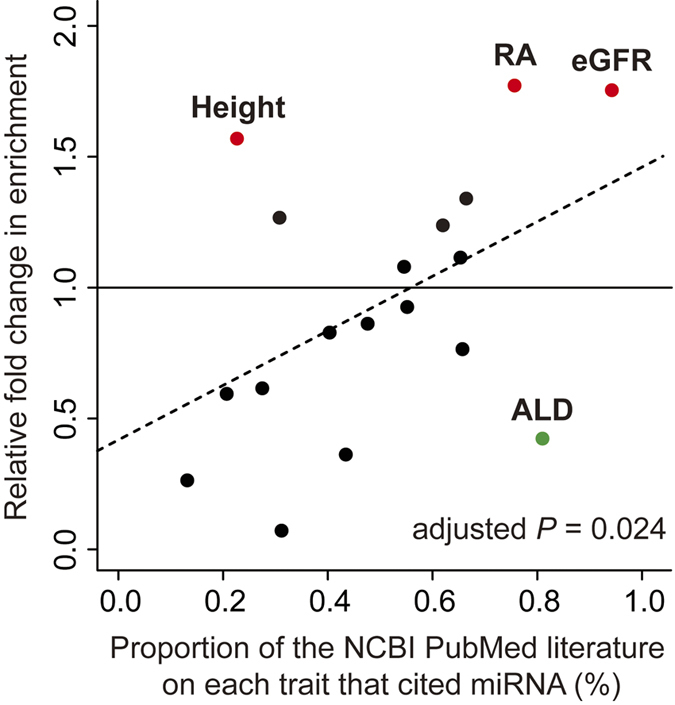
Relationships between literature-based knowledge about traits and the miRNA–target gene network enrichment in the GWAS. The proportion of the NCBI PubMed literature on each trait that cited miRNA in their context (*x*–axis) and relative fold changes in the enrichment of GWAS association signals on miRNA–target gene networks(= *F*_enrichment_; *y*–axis) indicated significant positive correlation (*P *= 0.024 adjusted with the number of the individuals in the original GWAS).

To validate the impact of the miRNA–target gene network as suggested by our MIGWAS method, we conducted a survey of miRNA citations in the existing literature to quantify our current knowledge of miRNA in the context of each trait as an independent resource with which to measure the impact of miRNA. On average, in the NCBI PubMed database, approximately 0.5% of literature on each trait cited miRNA (Supplementary Table 3). We observed significant positive correlations between the relative enrichment of miRNA–target gene association signals as estimated by MIGWAS (= *F*_enrichment_) and the proportions of the literature that cited miRNA (adjusted *P *= 0.024; Fig. 3). The highest citation proportion, 0.94%, was observed for eGFR, as suggested in previous biological studies^30^. This indicates that the impact of miRNA on human genetics, as suggested by our MIGWAS method, was also supported by the current knowledge on miRNA.

### Identification of therapeutic miRNAs that regulate drug target genes

As a feature, our method provides a list of miRNA and target gene pairs with excess genetic association signals. For the top three enrichment traits (RA, eGFR, and adult height), we highlighted 9, 6 and 25 miRNAs, respectively, and their target genes (both *P*_miRNA_ and *P*_gene_* *< 0.01 with high target prediction scores [top 1st percentile of the multiple algorithms]; Fig. 2, Table 1 and Supplementary Table 4). We found that some of the identified miRNA–target genes were also the drug target genes registered in the drug databases, including *DDX6*, *IFNAR1*, *PADI2*, and *FADS2* for RA, and *MMP24*, *PML*, and *SCN4A* for adult height. Considering utilities of these genes as therapeutic targets, the miRNAs and target gene pairs provided by our MIGWAS method should serve as an efficient screening resource for human genetics-driven novel drug discovery^28^,^31^,^32^. We note that miRNA targeting the larger numbers of genes could be likely to have higher power to be detected as candidates in our analytic pipeline, while these miRNAs could have a wide range of regulatory effects on gene expression profiles, and considered as promising candidates in terms of disease biology.

**Figure 4:**
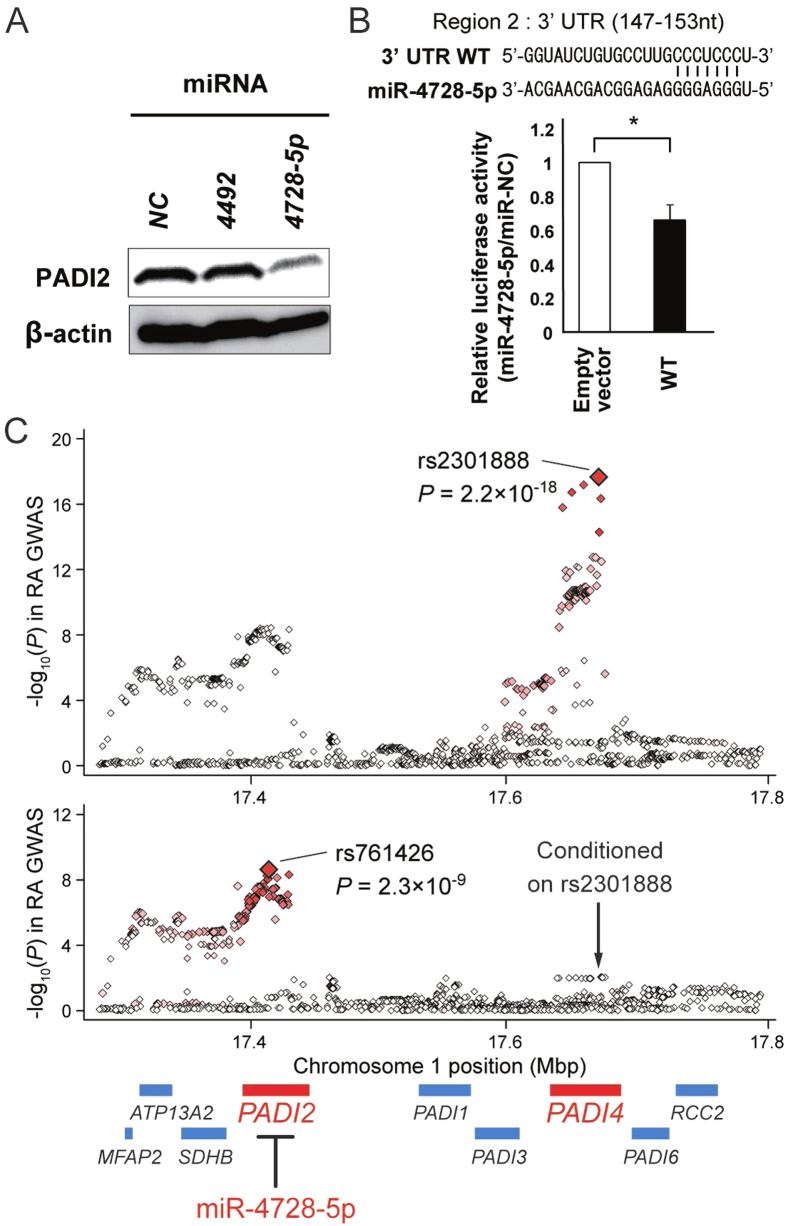
miR-4728-5p suppresses PADI2 protein expression, a novel RA risk gene as a potential therapeutic target. Western blotting (**A**) and luciferase assay (**B**) demonstrated that miR-4728-5p, the miRNA suggested by our MIGWAS analysis, suppresses PADI2 protein expression levels by direct binding to the 3′ UTR region (Supplementary Figures 2 and 3). Experiments were performed in triplicate, and each data point represents the mean (bars, SD). An asterisk represents Student’s *t*-test *P *< 0.05. (**C**) Conditional association analysis of the RA GWAS results^28^ in the *PADI2*–*PADI4* region. Each diamond represents the −log_10_ p-values of the SNPs. Red color for the diamond represents the *r*^*2*^ value with the most significantly associated SNP (larger red diamond). RefSeq genes are indicated below, and the gene nearest to the top-associated SNP is colored red. When conditioned on the top SNP at *PADI4* (rs2301888), an independent significant association was observed at *PADI2* (rs761426).

As an empirical example, we focused on *PADI2* at 1p36 pointed by multiple miRNAs (miR-4492 at 11q23 and miR-4728-5p at 17q12) in the context of the RA GWAS, as inhibition of this drug target gene is considered to be promising for treatment of autoimmune diseases^33^. We functionally confirmed that miR-4728-5p suppresses *PADI2* protein expression levels through direct binding to the 3′ UTR region. (Fig. 4A,B and Supplementary Figures 2 and 3). Being adjacently located at the well-known RA risk gene of *PADI4*, *PADI2* itself has not been recognized as a disease risk gene that satisfied the genome-wide significance threshold (*P *< 5.0 × 10^−8^)^28^,^34^. Motivated by its identification through our MIGWAS method, we conducted a conditional analysis of the *PADI4* locus with the top associated SNP in the GWAS meta-analysis (rs2301888, *P *= 2.2 × 10^−18^; Fig. 4C), and identified an independent significant association signal at *PADI2* (rs761426, adjusted *P *= 2.3 × 10^−9^). These findings suggest that our method can also contribute to the fine-mapping of causal genes embedded in GWAS results. The RA risk T allele of rs761426 has a cis-eQTL effect that increases *PADI2* mRNA expression in whole blood (*P *= 4.6 × 10^−12^; Supplementary Figure 4),^35^ thereby providing the possibility of the identified miRNA as a candidate of nucleic acid medicine. MiR-4728-5p was located in the previously reported RA risk loci at 17q12 (Supplementary Figure 5), while cis-eQTL effects of the regional SNPs on miR-4728-5p was not publicly available. We note that the RA risk SNPs in these loci were not located on the seed or target sequences of miR-4728-5p.

## Discussion

The integration of large-scale genetic studies with epigenomics resources should enhance our knowledge regarding human complex traits.^28^ In this study, our analytical method clearly indicated the significant impact of miRNA–target gene networks on the genetics of a variety of these traits. In particular, significant enrichment was observed for RA, eGFR, and adult height, as implicated by the previous biological studies.^2^,^30^ Our method also provided a list of miRNA and target gene pairs with excess genetic association signals, which could contribute to fine-mapping of causal genes and the screening of therapeutic miRNAs and drug target genes^28^,^31^,^32^. As an empirical example, we identified *PADI2* as a novel risk gene of RA that could be a potential therapeutic target, as well as the miRNA that suppresses PADI2 protein expression (miR-4728-5p)^2^,^30^. Such framework integrating disease genetics and network-based information could be utilized for predicting clinical phenotypes as a future research strategy^36^.

Although the biological importance of miRNA in disease etiologies has long been suggested, strategies linking miRNA with disease genetics have made less progress than other epigenomics resources such as mRNA expression profiles. We show here that our newly developed method can bridge this missing link by validating the simple hypothesis that the miRNA and target gene pairs implicated in disease biology will likely exhibit enrichment of association signals in GWAS results. This integrative approach, which included multiple miRNA–target gene prediction algorithms and variable prediction score cutoff thresholds, achieved an unbiased interpretation of target prediction uncertainties. Additional implementation to estimate variance of the *F*_enrichment_ and *P*_enrichment_ would be informative to validate robustness of our integrative approach. Further application of our MIGWAS method to the results of the GWASs of additional disorders is warranted.

We found that estimated impact of miRNA on human genetics was consistent with the literature-based current knowledge of miRNA on these traits. While our literature-based analysis did not involve manual curation of the texts, which can weaken the evidence, the observed concordance might provide a clue to prioritize the diseases to be assessed.

We note that the detailed biological mechanisms by which the variants located in each miRNA and target gene region confer disease risk are yet to be elucidated. Considering the essential roles of miRNAs in the regulation of target gene expression, further accumulation of miRNA eQTL studies, particularly those focusing on the effect of trans-miRNA eQTL on potential target genes,^7^ should clarify these mechanisms. Whereas the existing microarray-based miRNA expression assays evaluate limited numbers of miRNAs, recent advances in the RNA-seq technology should provide expression profiles of wide ranges of functional miRNAs.

In summary, our study demonstrated the significant impact of miRNA–target gene networks on the genetics of human complex traits. This information should contribute to our understanding of the roles of miRNA in disease etiology and the potential uses in drug discovery.

## Methods

### Collection of GWAS results of human complex traits

We collected summary statistics (p-values) of the previously published GWASs of human complex traits from public web sites or collaborators. To ensure the statistical power of our analysis, we restricted GWASs to those including >30,000 individuals. For GWASs that reported multiple traits in the same paper, we did not redundantly add the numbers of individuals. We collected GWAS results of 18 human complex traits that comprised of 1,765,016 individuals in total; adult height^15^, age at menarche (AAM)^29^, age-related macular degeneration (AMD)^27^, Alzheimer’s disease (ALD)^25^, blood pressure (diastolic [DBP] and systolic [SBP])^21^, body mass index (BMI)^16^, bone mineral density (femoral; BMD)^22^, estimated glomerular filtration rate based on serum creatinine (eGFR)^20^, high-density lipoprotein (HDL)^19^, low-density lipoprotein (LDL)^19^, triglyceride (TG)^19^, platelet counts (PLT)^17^, red blood cell counts (RBC)^18^, rheumatoid arthritis (RA)^28^, schizophrenia (SCZ)^26^, type II diabetes mellitus (T2D)^23^, and uric acid (UA; Supplementary Table 1)^24^. SNP information was re-assigned based on the UCSC hg19 reference.

As a negative control, we generated in total five null GWAS results (datasets 1–5), using 1000 Genomes Project Phase I (α) European genotype data. We applied SNP quality control filters as described elsewhere^28^ and randomly divided the individuals into case-control groups (case:control ratio* *= 1:1). We then conducted a GWAS using a logistic regression analysis implemented in PLINK v1.90.

### Translation of SNP-based association signals into gene- or miRNA-based signals

To evaluate the association signals of human complex traits with the respective genes and miRNAs, we translated the genome-wide SNP p-values of each GWAS into a set of gene-based or miRNA-based p-values (= *P*_gene_ or *P*_miRNA_), adjusted according to local linkage disequilibrium structures and gene or miRNA sizes. We used the MAGENTA software to conduct this step^10^. Gene information was obtained from the UCSC hg19 reference and miRNA information was obtained from miRBase release 20^ ^3. We excluded genes and miRNAs located in the major histocompatibility complex (MHC) region at 6p21.3^37^,^38^,^39^, while inclusion or exclusion of the MHC region did not change the analytic results substantially (data not shown).

For the gene or miRNA p-values obtained from the null GWAS, we artificially induced inflation of the association signals of the null dataset 1, by inversely applying genomic control (GC) corrections with λ_GC_ values to *P*_gene_ and *P*_miRNA_ in the ranges of 1.0 (i.e., no changes in p-values after correction) to 3.0 (i.e., strong inflation induced after correction).

### Curation of miRNA–target gene network information

We downloaded miRNA–target gene prediction scores calculated using the major target prediction algorithms on January 31st, 2015 (*n *= 4; miRDB^11^, MiRmap^12^, PITA^13^, and TargetScan^14^; Supplementary Table 2). The methodological comparisons of these prediction algorithms have been discussed elsewhere^9^. We assigned miRNA and gene information according to miRBase (release 20)^3^ and the UCSC hg19 reference, respectively. We excluded closely located miRNA and gene pairs (defined as physical distances between the miRNA and gene below 1 Mbp), as the genome-wide association signals of such miRNAs and genes could be non-independent because of local linkage disequilibrium.

### Enrichment analysis of association signals in the miRNA–target gene pairs

We hypothesized that genetic association signals are relatively enriched in the miRNA and gene pairs, regarding traits for which miRNA plays important etiological roles. To empirically test this hypothesis, we evaluated whether association signals of both of the miRNA and target gene pairs suggested by the miRNA–target gene prediction algorithms with defined score thresholds were more likely to demonstrate significant associations than that would be expected by chance. To robustly estimate the degree of enrichment, we integrated the analysis results from various score thresholds of multiple prediction algorithms.

Let 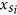 be the set of miRNA and target gene pairs that satisfies the prediction score threshold calculated using the *i* th prediction algorithm (= *s*_*i*_), and 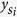 be the subset of 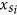 for which both the *P*_miRNA_ and *P*_gene_ satisfy the nominal association threshold (α* *= 0.01). We defined 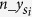/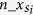 as a metric to represent the association signal enrichment of miRNA and target gene pairs, where 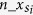 and 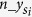 represent the numbers of miRNA and target gene pairs included in 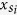 and 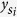, respectively. We estimated the null distribution of this metric using a permutation procedure (×10,000 iterations). For each iteration step, we randomly shuffled the miRNA and target gene pair connections within 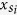, and generated dummy sets of 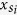
*’ and* 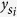
’ as described above. We shuffled miRNA-target gene pairs by permuting pair labels within all the pair collections of 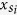, and thus, equal weight was assigned to each pair but not to each miRNA or each target gene. We defined a relative fold change in the metric 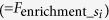 as (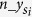/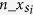)/*m*(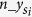
’/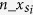
’), where *m*(*t*) represents the mean value of the distribution *t*. The significance of the metric 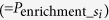 was evaluated using a one-sided permutation test in its null distribution.

We then sequentially integrated 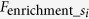 or 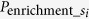 by sliding the threshold values of *s*_*i*_ from the top 10th percentile to the 0.1th percentile of the prediction score distribution on a logarithm scale with a number of partition* *= 8. Considering that estimation of 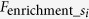 or 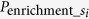 can be biased when distributions of 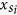
*’ and* 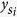
’ are sparse, we only integrated the results obtained under the condition of *m*(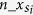
’) ≥ 5. Finally, we integrated the results of the multiple prediction algorithms (*n*_algorithm_* *= 4), by averaging the fold change estimates and meta-analyzing the enrichment significance. Namely, we estimated the overall fold change in enrichment, *F*_enrichment_, and significance of enrichment, *P*_enrichment_, as,


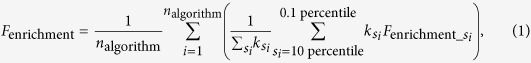


and


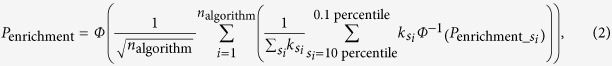


where 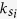
* *= 1 when *m*(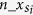
’) ≥ 5 and 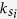
* *= 0 when *m*(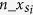
’)* *< 5. We note that we did not observe the condition when 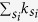
* *= 0. *Φ* represents the cumulative distribution function (*c.d.f.*) of the normal distribution. The source codes for MIGWAS and the data resources are available upon request to the authors.

### Survey of miRNA citations in human complex trait literature

To relatively quantify our current knowledge about miRNA in human complex traits, we conducted a survey of miRNA citations in previously published literatures concerning the 18 human complex traits that we examined. We calculated the proportions of literature concerning each trait that cited miRNA in their contexts according to a search of the NCBI PubMed database on July 31st, 2015. Considering that most of the miRNA literatures have been published in recent years, we confined our analysis to those published in the last 5 years (2010–2014). The proportions were calculated as follows:


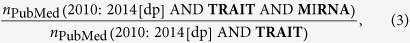


where *n*_PubMed_(*x*) represents the number of the literatures obtained from the NCBI PubMed database when using *x* as a search term. **MIRNA** was a miRNA-specific search term defied as “(miRNA OR miRNAs OR microRNA NOT mirna[au])”. **TRAIT** was a search term used for each trait and is defined in detail in Supplementary Table 3. We note that for the traits representing quantitative values, we additionally included the disorders defined as extreme trait values in **TRAIT** (e.g., gout for uric acid), as these traits and disorder are likely to share biological and genetic backgrounds and have often been examined together.^24^

The association between *F*_enrichment_ and the calculated proportions was evaluated through a linear regression analysis. To account for potential heterogeneity in the statistical powers of the original GWAS that might affect *F*_enrichment_, we included the numbers of the individuals in the GWASs as a covariate.

### List of the miRNA and target gene pairs with association signal enrichment

For the human complex traits that demonstrated significant enrichment of the genetic association signals in the miRNA–target gene networks after Bonferroni correction (*P*_*enrichment*_* *< 0.05/18* *= 0.0028), we made a list of the miRNA and target gene pairs. We selected the pairs if (i) both *P*_miRNA_ and *P*_gene_ satisfied the nominal association threshold (α* *= 0.01), and (ii) the prediction scores of the pairs were ranked within the top 1 percentile in multiple prediction algorithms.

We next annotated the selected miRNA–targeted genes based on whether they had been registered as therapeutic drug target genes. We used the SuperTarget database^40^ and a previously curated drug target gene database^28^ based on the DrugBank^41^ and Therapeutic Targets Database^42^.

### Western blotting

The following primary antibodies were used for western blotting: anti-PADI2 (12110-1-AP) (Proteintech, Chicago, IL) and anti-β-actin (Sigma, St. Louis, MO). Western blotting was performed as described elsewhere^43^.

### Luciferase assay of miRNAs that target disease risk gene

HeLa and MCF7 cell lines were maintained in DMEM containing 10% fetal bovine serum (FBS). Luciferase reporter plasmids were constructed by inserting the 3′ UTR of *PADI2* (regions 1–8) downstream of the luciferase gene within the pmirGLO Dual-Luciferase miRNA Target Expression Vector (Promega, Madison, WI). Luciferase reporter plasmids and 10 nmol/L of *miRNAs* (*miR-Negative Control* [*NC*], *miR-4429* or *miR-4728-5p*; Thermo Fisher Scientific, Waltham, MA) were co-transfected in HeLa cells using Lipofectamine 2000 (Thermo Fisher Scientific) according to the manufacturer’s instrument. Forty-eight hours after transfection with luciferase reporter plasmids and miRNAs, Firefly and Renilla, as an internal control, luciferase activity were measured by the Dual-Luciferase Reporter Assay System (Promega). Relative luciferase activity was calculated by normalizing Firefly luciferase activity by its corresponding Renilla luciferase activity. Supplementary Figures 2 and 3 showed the sequences of primers for constructing of each luciferase reporter plasmid. Experiments were performed in triplicate, and each data point represents the mean (bars, SD). Student’s *t*-test was used for statistical analysis.

### Conditional association analysis of the GWAS results

A conditional association analysis of the RA GWAS meta-analysis summary statistics was conducted with respect to the *PADI2*–*PADI4* region using GCTA software^44^. We performed a conditional analysis separately for the European and Asian GWAS results, and subsequently meta-analyzed the conditioned results using the inverse-variance method. Cis-eQTL analysis results of the SNP was obtained from Genotype-Tissue Expression (GTEx) Analysis Release v4 (dbGaP Accession phs000424.v4.p1)^35^.

## Additional Information

**How to cite this article**: Okada, Y. *et al.* Significant impact of miRNA-target gene networks on genetics of human complex traits. *Sci. Rep.* **6**, 22223; doi: 10.1038/srep22223 (2016).

## Supplementary Material

Supplementary Information

## Figures and Tables

**Table 1:** miRNAs and target genes listed by the MIGWAS analysis.

Trait	miRNAs enriched in the GWAS results with their target genes^a^	*P*_enrichment_
RA	**miR-130b-3p** (*DDX6*), miR-638, miR-762, **miR-3155a** (*IFNAR1*),miR-3155b, miR-3202, miR-3714, **miR-4492** (*PADI2*), **miR-4728-5p**(*PADI2*, *FADS2*)	1.7 × 10^−4^
eGFR	miR-661, miR-2355-5p, miR-4313, miR-4487,miR-4672, miR-4728-5p	3.6 × 10^−4^
Adult height	let-7a-5p, let-7d-5p, miR-7-1-3p, miR-15a-5p, miR-17-5p,miR-20a-5p, miR-30d-3p, miR-146b-3p, miR-217, **miR-608** (*MMP24*, *PML*), **miR-940** (*SCN4A*), miR-629-3p,miR-1225-3p, miR-1225-5p, miR-1227-5p, miR-3120-3p,miR-3613-3p, miR-3675-5p, miR-4419a, miR-4487,miR-4489, miR-4690-3p, miR-4690-5p, miR-4713-5p, miR-4722-3p	5.2 × 10^−4^

^a^miRNAs are indicated for significantly enriched traits suggested using the MIGWAS method. MiRNAs that target known drug target gene(s) are underscored with bars, with parentheses indicating the target(s). Full miRNA and target gene lists are provided in Supplementary Table 4.
