# Supplementary material for: Significant impact of miRNA–target gene networks on genetics of human complex traits
Source: Sci Rep. 2016 Mar 1;6:22223. doi: 10.1038/srep22223 (PMC4772006; doi:10.1038/srep22223)
Supplement: Supplementary Information [file srep22223-s1.pdf]

Significant impact of miRNA–target gene networks on genetics of human complex traits

Yukinori Okada*, Tomoki Muramatsu, Naomasa Suita, Masahiro Kanai, Eiryo Kawakami, Valentina Iotchkova, Nicole Soranzo, Johji Inazawa, Toshihiro Tanaka.

*Corresponding author:

Yukinori Okada, MD, PhD

E-mail: yokada@riken.jp

Supplementary Table 1. Summary of the GWAS results of human complex traits

Human complex trait	No. Samples	No. SNPs in GWAS	Ref.
Adult height	253,288	2,550,858	1
Age at menarche (AAM)	182,416	2,441,815	2
Age-related macular degeneration (AMD)	56,494	2,399,382	3
Alzheimer's disease (ALD)	54,162	7,055,881	4
Blood pressure (diastolic; DBP)<br>Blood pressure (systolic; SBP)	69,395	2,673,126<br>2,650,287	5
Body mass index (BMI)	339,224	2,555,510	6
Bone mineral density (femoral; BMD)	32,961	2,554,591	7
Estimated glomerular filtration rate based on serum creatinine by creatinine (eGFR)	67,093	2,775,224	8
High-density lipoprotein (HDL)<br>Low-density lipoprotein (LDL)<br>Triglyceride (TG)	188,577	2,446,638<br>2,436,956<br>2,438,636	9
Platelet counts (PLT)	66,867	2,703,393	10
Red blood cell counts (RBC)	71,861	2,589,454	11
Rheumatoid arthritis (RA)	80,799	9,739,303	12
Schizophrenia (SCZ)	81,080	9,444,230	13
Type II diabetes mellitus (T2D)	110,452	2,915,011	14
Uric acid (UA)	110,347	2,207,610	15
Total	1,765,016	-	-

Supplementary Table 2. Characteristics of the miRNA–target gene prediction databases

Database	No. miRNAs	No. target genes	No. miRNA–target gene pairs	Ref.
miRDB	2,578	16,638	902,439	16
miRmap	1,929	17,933	10,569,659	17
PITA	586	14,027	2,900,809	18
TargetScan	1,514	17,795	3,339,730	19

Supplementary Table 3. Search terms of the traits for NCBI PubMed database and results of the literature search

Human Complex trait	Search term for NCBI PubMed database	No. PubMed citations for		Proportion
		miRNA and trait	Trait	
Adult height	height OR body size OR stature	299	133,137	0.225%
Age at menarche	age at menarche OR menarche	9	1,655	0.544%
Age-related macular degeneration	age-related macular degenerations	25	8,075	0.310%
Alzheimer's disease	Alzheimer's disease OR Alzheimer	295	36,511	0.808%
Blood pressure (diastolic)	blood pressure OR hypertension	391	143,297	0.273%
Blood pressure (systolic)	blood pressure OR hypertension	391	82,474	0.474%
Body mass index	body mass index OR body weight	317	154,747	0.205%
Bone mineral density (femoral)	bone mineral density OR osteoporosis	97	31,707	0.306%
eGFR by creatinine	glomerular filtration rate OR renal function OR kidney function	947	100,697	0.940%
High-density lipoprotein	high-density lipoprotein	117	17,958	0.652%
Low-density lipoprotein	low-density lipoprotein	136	20,530	0.662%
Platelet counts	platelet	250	40,462	0.618%
Red blood cell counts	red blood cell OR erythrocyte OR anemia	249	57,578	0.432%
Rheumatoid arthritis	rheumatoid arthritis	171	22,657	0.755%
Schizophrenia	schizophrenia	169	25,793	0.655%
Triglyceride	triglyceride	109	27,145	0.402%
Type II diabetes mellitus	type 2 diabetes OR type II diabetes	273	49,661	0.550%
Uric acid	uric acid OR urate OR gout	11	8,486	0.130%

Supplementary Table 4. miRNAs and target genes listed by the MIGWAS analysis

Trait	miRNA					Target gene		
	Name (mature)	ID	Name	ID	P_{miRNA}	Name	ID	P_{gene}
RA	hsa-miR-638	MIMAT0003308	hsa-mir-638	MI0003653	2.9.E-06	SLC25A23	79085	7.3.E-04
	hsa-miR-762	MIMAT0010313	hsa-mir-762	MI0003892	5.2.E-04	PHLDB1	23187	4.2.E-05
	hsa-miR-4728-5p	MIMAT0019849	hsa-mir-4728	MI0017365	1.5.E-09	HCFC1	3054	3.2.E-14
	hsa-miR-4728-5p	MIMAT0019849	hsa-mir-4728	MI0017365	1.5.E-09	TMUB2	79089	6.7.E-03
	hsa-miR-3714	MIMAT0018165	hsa-mir-3714	MI0016135	4.3.E-06	PAPOLG	64895	1.3.E-05
	hsa-miR-3202	MIMAT0015089	hsa-mir-3202-2	MI0014253	2.7.E-14	PDGFB	5155	3.8.E-10
	hsa-miR-4728-5p	MIMAT0019849	hsa-mir-4728	MI0017365	1.5.E-09	KHSRP	8570	7.7.E-04
	hsa-miR-4728-5p	MIMAT0019849	hsa-mir-4728	MI0017365	1.5.E-09	RAD51L1	5890	3.4.E-05
	hsa-miR-3925-3p	MIMAT0019228	hsa-mir-3925	MI0016433	8.2.E-03	PHTF1	10745	1.2.E-14
	hsa-miR-4728-5p	MIMAT0019849	hsa-mir-4728	MI0017365	1.5.E-09	AGAP2	116986	3.0.E-04
	hsa-miR-4728-5p	MIMAT0019849	hsa-mir-4728	MI0017365	1.5.E-09	PA2G4	5036	5.6.E-07
	hsa-miR-4728-5p	MIMAT0019849	hsa-mir-4728	MI0017365	1.5.E-09	SYNGR1	9145	1.9.E-10
	hsa-miR-4492	MIMAT0019027	hsa-mir-4492	MI0016854	3.0.E-12	TMEM151B	441151	5.8.E-14
	hsa-miR-4492	MIMAT0019027	hsa-mir-4492	MI0016854	3.0.E-12	STAC2	342667	2.9.E-04
	hsa-miR-4728-5p	MIMAT0019849	hsa-mir-4728	MI0017365	1.5.E-09	POU2AF1	5450	3.0.E-03
	hsa-miR-4728-5p	MIMAT0019849	hsa-mir-4728	MI0017365	1.5.E-09	FADS2	9415	1.6.E-03
	hsa-miR-4728-5p	MIMAT0019849	hsa-mir-4728	MI0017365	1.5.E-09	C11orf9	745	1.3.E-03
	hsa-miR-4728-5p	MIMAT0019849	hsa-mir-4728	MI0017365	1.5.E-09	DAGLA	747	1.8.E-03
	hsa-miR-4728-5p	MIMAT0019849	hsa-mir-4728	MI0017365	1.5.E-09	VPS37C	55048	5.1.E-03
	hsa-miR-762	MIMAT0010313	hsa-mir-762	MI0003892	5.2.E-04	MECP2	4204	3.2.E-14
	hsa-miR-3155b	MIMAT0019012	hsa-mir-3155b	MI0016839	4.3.E-03	HIPK1	204851	2.3.E-14
	hsa-miR-3155a	MIMAT0015029	hsa-mir-3155a	MI0014183	5.8.E-06	HIPK1	204851	2.3.E-14
	hsa-miR-762	MIMAT0010313	hsa-mir-762	MI0003892	5.2.E-04	TSPAN33	340348	6.4.E-08
	hsa-miR-4492	MIMAT0019027	hsa-mir-4492	MI0016854	3.0.E-12	SON	6651	6.6.E-04
	hsa-miR-762	MIMAT0010313	hsa-mir-762	MI0003892	5.2.E-04	TMEM151B	441151	5.8.E-14
	hsa-miR-4492	MIMAT0019027	hsa-mir-4492	MI0016854	3.0.E-12	PADI2	11240	1.2.E-05
	hsa-miR-130b-3p	MIMAT0000691	hsa-mir-130b	MI0000748	2.8.E-03	DDX6	1656	4.7.E-12
	hsa-miR-3925-3p	MIMAT0019228	hsa-mir-3925	MI0016433	8.2.E-03	ZNF594	84622	7.5.E-04
	hsa-miR-4728-5p	MIMAT0019849	hsa-mir-4728	MI0017365	1.5.E-09	SLC29A1	2030	5.2.E-14
	hsa-miR-3155a	MIMAT0015029	hsa-mir-3155a	MI0014183	5.8.E-06	IFNAR1	3454	4.9.E-03
	hsa-miR-3202	MIMAT0015089	hsa-mir-3202-2	MI0014253	2.7.E-14	RAB5B	5869	4.9.E-07
	hsa-miR-3202	MIMAT0015089	hsa-mir-3202-2	MI0014253	2.7.E-14	POU2AF1	5450	3.0.E-03
	hsa-miR-4728-5p	MIMAT0019849	hsa-mir-4728	MI0017365	1.5.E-09	UTP11L	51118	3.2.E-04
	hsa-miR-4728-5p	MIMAT0019849	hsa-mir-4728	MI0017365	1.5.E-09	PADI2	11240	1.2.E-05
	hsa-miR-4728-5p	MIMAT0019849	hsa-mir-4728	MI0017365	1.5.E-09	C1orf93	127281	3.4.E-11
eGFR	hsa-miR-4728-5p	MIMAT0019849	hsa-mir-4728	MI0017365	7.4.E-04	LRP1	4035	3.6.E-03
	hsa-miR-4672	MIMAT0019754	hsa-mir-4672	MI0017303	3.2.E-03	ACVR2A	92	2.5.E-03
	hsa-miR-2355-5p	MIMAT0016895	hsa-mir-2355	MI0015873	4.2.E-03	AMIGO1	57463	1.1.E-06
	hsa-miR-661	MIMAT0003324	hsa-mir-661	MI0003669	1.9.E-03	AP2A2	161	3.5.E-03
	hsa-miR-661	MIMAT0003324	hsa-mir-661	MI0003669	1.9.E-03	MUC6	4588	3.5.E-03
	hsa-miR-4313	MIMAT0016865	hsa-mir-4313	MI0015843	1.4.E-09	MBD6	114785	1.4.E-04
	hsa-miR-4728-5p	MIMAT0019849	hsa-mir-4728	MI0017365	7.4.E-04	ABCG4	64137	5.6.E-03
	hsa-miR-661	MIMAT0003324	hsa-mir-661	MI0003669	1.9.E-03	SMPD3	55512	3.1.E-04
	hsa-miR-2355-5p	MIMAT0016895	hsa-mir-2355	MI0015873	4.2.E-03	CELF1	10658	6.3.E-03
	hsa-miR-4756-5p	MIMAT0019899	hsa-mir-4756	MI0017397	7.4.E-03	ABCG4	64137	5.6.E-03
	hsa-miR-4756-5p	MIMAT0019899	hsa-mir-4756	MI0017397	7.4.E-03	HYOU1	10525	7.5.E-03
	hsa-miR-4487	MIMAT0019021	hsa-mir-4487	MI0016848	3.0.E-03	PSMA5	5686	1.7.E-07
	hsa-miR-4487	MIMAT0019021	hsa-mir-4487	MI0016848	3.0.E-03	SORT1	6272	3.7.E-07
	hsa-miR-661	MIMAT0003324	hsa-mir-661	MI0003669	1.9.E-03	LIF	3976	3.9.E-03
	hsa-miR-2355-5p	MIMAT0016895	hsa-mir-2355	MI0015873	4.2.E-03	STAC2	342667	2.5.E-05
	hsa-miR-4756-5p	MIMAT0019899	hsa-mir-4756	MI0017397	7.4.E-03	LASS2	29956	6.0.E-08

Trait	miRNA					Target gene		
	Name (mature)	ID	Name	ID	P_{miRNA}	Name	ID	P_{gene}
Height	hsa-miR-608	MIMAT0003276	hsa-mir-608	MI0003621	3.8.E-03	MKNK2	2872	1.5.E-03
	hsa-miR-608	MIMAT0003276	hsa-mir-608	MI0003621	3.8.E-03	PITPNM2	57605	2.9.E-03
	hsa-miR-608	MIMAT0003276	hsa-mir-608	MI0003621	3.8.E-03	FAM101A	144347	1.5.E-03
	hsa-miR-4492	MIMAT0019027	hsa-mir-4492	MI0016854	6.2.E-03	TRMT61A	115708	5.2.E-03
	hsa-let-7d-5p	MIMAT0000065	hsa-let-7d	MI0000065	1.6.E-03	LIN28B	389421	2.0.E-03
	hsa-miR-608	MIMAT0003276	hsa-mir-608	MI0003621	3.8.E-03	TNS4	84951	1.5.E-03
	hsa-miR-20a-5p	MIMAT0000075	hsa-mir-20a	MI0000076	4.0.E-03	ZNFX1	57169	1.9.E-03
	hsa-miR-17-5p	MIMAT0000070	hsa-mir-17	MI0000071	4.0.E-03	ZNFX1	57169	1.9.E-03
	hsa-miR-608	MIMAT0003276	hsa-mir-608	MI0003621	3.8.E-03	MMP24	10893	1.9.E-03
	hsa-miR-608	MIMAT0003276	hsa-mir-608	MI0003621	3.8.E-03	ACTR1A	10121	2.1.E-03
	hsa-miR-4722-5p	MIMAT0019836	hsa-mir-4722	MI0017357	2.0.E-03	NFAT5	10725	8.5.E-03
	hsa-miR-1225-3p	MIMAT0005573	hsa-mir-1225	MI0006311	2.2.E-03	CDT1	81620	2.1.E-03
	hsa-miR-4487	MIMAT0019021	hsa-mir-4487	MI0016848	2.3.E-03	SUFU	51684	2.6.E-03
	hsa-miR-608	MIMAT0003276	hsa-mir-608	MI0003621	3.8.E-03	CELF1	10658	2.8.E-03
	hsa-miR-608	MIMAT0003276	hsa-mir-608	MI0003621	3.8.E-03	PTCH1	5727	2.1.E-03
	hsa-miR-15a-5p	MIMAT0000068	hsa-mir-15a	MI0000069	2.2.E-03	PPM1A	5494	1.8.E-03
	hsa-miR-4492	MIMAT0019027	hsa-mir-4492	MI0016854	6.2.E-03	ZMIZ1	57178	1.7.E-03
	hsa-miR-940	MIMAT0004983	hsa-mir-940	MI0005762	1.6.E-03	SCN4A	6329	1.6.E-03
	hsa-miR-3675-5p	MIMAT0018098	hsa-mir-3675	MI0016076	2.4.E-03	FEV	54738	1.7.E-03
	hsa-miR-4323	MIMAT0016875	hsa-mir-4323	MI0015853	8.3.E-03	PIP4K2B	8396	4.1.E-03
	hsa-miR-608	MIMAT0003276	hsa-mir-608	MI0003621	3.8.E-03	SETD8	387893	2.6.E-03
	hsa-miR-4492	MIMAT0019027	hsa-mir-4492	MI0016854	6.2.E-03	TMEM90A	646658	1.3.E-03
	hsa-miR-4487	MIMAT0019021	hsa-mir-4487	MI0016848	2.3.E-03	KHNYN	23351	1.2.E-03
	hsa-let-7a-5p	MIMAT0000062	hsa-let-7a-1	MI0000060	1.6.E-03	LIN28B	389421	2.0.E-03
	hsa-miR-608	MIMAT0003276	hsa-mir-608	MI0003621	3.8.E-03	CREB5	9586	2.9.E-03
	hsa-miR-548aq-5p	MIMAT0022263	hsa-mir-548aq	MI0019130	1.2.E-03	CDK6	1021	2.5.E-03
	hsa-miR-15a-5p	MIMAT0000068	hsa-mir-15a	MI0000069	2.2.E-03	PAPPA	5069	2.0.E-03
	hsa-miR-1225-3p	MIMAT0005573	hsa-mir-1225	MI0006311	2.2.E-03	STAT2	6773	4.2.E-03
	hsa-miR-940	MIMAT0004983	hsa-mir-940	MI0005762	1.6.E-03	CS	1431	3.2.E-03
	hsa-miR-4713-5p	MIMAT0019820	hsa-mir-4713	MI0017347	2.8.E-03	MTCH2	23788	2.5.E-03
	hsa-miR-4690-5p	MIMAT0019779	hsa-mir-4690	MI0017323	3.2.E-03	TULP4	56995	4.6.E-03
	hsa-miR-629-3p	MIMAT0003298	hsa-mir-629	MI0003643	6.3.E-04	TP53INP2	58476	2.6.E-03
	hsa-miR-608	MIMAT0003276	hsa-mir-608	MI0003621	3.8.E-03	PDLIM4	8572	1.5.E-03
	hsa-miR-608	MIMAT0003276	hsa-mir-608	MI0003621	3.8.E-03	PML	5371	2.0.E-03
	hsa-miR-608	MIMAT0003276	hsa-mir-608	MI0003621	3.8.E-03	ISLR2	57611	1.6.E-03
	hsa-miR-146b-3p	MIMAT0004766	hsa-mir-146b	MI0003129	2.7.E-03	ZSCAN2	54993	2.2.E-03
	hsa-miR-4690-3p	MIMAT0019780	hsa-mir-4690	MI0017323	3.2.E-03	SUPT3H	8464	4.3.E-03
	hsa-miR-4722-3p	MIMAT0019837	hsa-mir-4722	MI0017357	2.0.E-03	SYT12	91683	4.4.E-03
	hsa-miR-4492	MIMAT0019027	hsa-mir-4492	MI0016854	6.2.E-03	TNS4	84951	1.5.E-03
	hsa-miR-629-3p	MIMAT0003298	hsa-mir-629	MI0003643	6.3.E-04	PAPPA	5069	2.0.E-03
	hsa-miR-4690-3p	MIMAT0019780	hsa-mir-4690	MI0017323	3.2.E-03	NMT1	4836	1.9.E-03
	hsa-miR-4487	MIMAT0019021	hsa-mir-4487	MI0016848	2.3.E-03	MLLT6	4302	5.9.E-03
	hsa-miR-4419a	MIMAT0018931	hsa-mir-4419a	MI0016755	2.5.E-03	ABCB8	11194	7.6.E-03
	hsa-miR-7-1-3p	MIMAT0004553	hsa-mir-7-1	MI0000263	4.6.E-03	USP37	57695	2.9.E-03
	hsa-miR-3613-3p	MIMAT0017991	hsa-mir-3613	MI0016003	2.3.E-03	TULP4	56995	4.6.E-03
	hsa-miR-4492	MIMAT0019027	hsa-mir-4492	MI0016854	6.2.E-03	CABLES1	91768	2.1.E-03
	hsa-miR-1225-5p	MIMAT0005572	hsa-mir-1225	MI0006311	2.2.E-03	CDC14B	8555	2.4.E-03
	hsa-miR-608	MIMAT0003276	hsa-mir-608	MI0003621	3.8.E-03	KCNJ12	3768	8.6.E-03
	hsa-miR-608	MIMAT0003276	hsa-mir-608	MI0003621	3.8.E-03	CISD3	284106	4.9.E-03
	hsa-miR-608	MIMAT0003276	hsa-mir-608	MI0003621	3.8.E-03	PCGF2	7703	5.5.E-03
	hsa-miR-608	MIMAT0003276	hsa-mir-608	MI0003621	3.8.E-03	ACBD4	79777	1.8.E-03
	hsa-miR-608	MIMAT0003276	hsa-mir-608	MI0003621	3.8.E-03	MAP3K3	4215	2.8.E-03
	hsa-miR-608	MIMAT0003276	hsa-mir-608	MI0003621	3.8.E-03	LIMD2	80774	2.5.E-03
	hsa-miR-4492	MIMAT0019027	hsa-mir-4492	MI0016854	6.2.E-03	NFIC	4782	1.8.E-03
	hsa-miR-4690-3p	MIMAT0019780	hsa-mir-4690	MI0017323	3.2.E-03	DNAJB2	3300	2.0.E-03
	hsa-miR-608	MIMAT0003276	hsa-mir-608	MI0003621	3.8.E-03	NFIC	4782	1.8.E-03
	hsa-miR-4489	MIMAT0019023	hsa-mir-4489	MI0016850	3.2.E-03	TP53INP2	58476	2.6.E-03
	hsa-miR-1227-5p	MIMAT0022941	hsa-mir-1227	MI0006316	1.5.E-03	GPSM1	26086	2.2.E-03
	hsa-miR-4419a	MIMAT0018931	hsa-mir-4419a	MI0016755	2.5.E-03	KDM2A	22992	5.7.E-03
	hsa-miR-4492	MIMAT0019027	hsa-mir-4492	MI0016854	6.2.E-03	PADI2	11240	1.5.E-03
	hsa-miR-940	MIMAT0004983	hsa-mir-940	MI0005762	1.6.E-03	PCBD2	84105	2.1.E-03
	hsa-miR-30d-3p	MIMAT0004551	hsa-mir-30d	MI0000255	1.7.E-03	STAU1	6780	2.7.E-03
	hsa-miR-217	MIMAT0000274	hsa-mir-217	MI0000293	1.3.E-03	PPM1A	5494	1.8.E-03
	hsa-miR-15a-5p	MIMAT0000068	hsa-mir-15a	MI0000069	2.2.E-03	LUZP1	7798	2.4.E-03
	hsa-miR-548aq-5p	MIMAT0022263	hsa-mir-548aq	MI0019130	1.2.E-03	NFAT5	10725	8.5.E-03
	hsa-miR-4419a	MIMAT0018931	hsa-mir-4419a	MI0016755	2.5.E-03	NYNRIN	57523	1.4.E-03
	hsa-miR-4419a	MIMAT0018931	hsa-mir-4419a	MI0016755	2.5.E-03	LASP1	3927	4.6.E-03
	hsa-miR-3120-3p	MIMAT0014982	hsa-mir-3120	MI0014136	1.5.E-03	RBM12	10137	2.3.E-03

Supplementary Figure 1. Null GWAS data indicated no association signal enrichment in miRNA–target gene networks

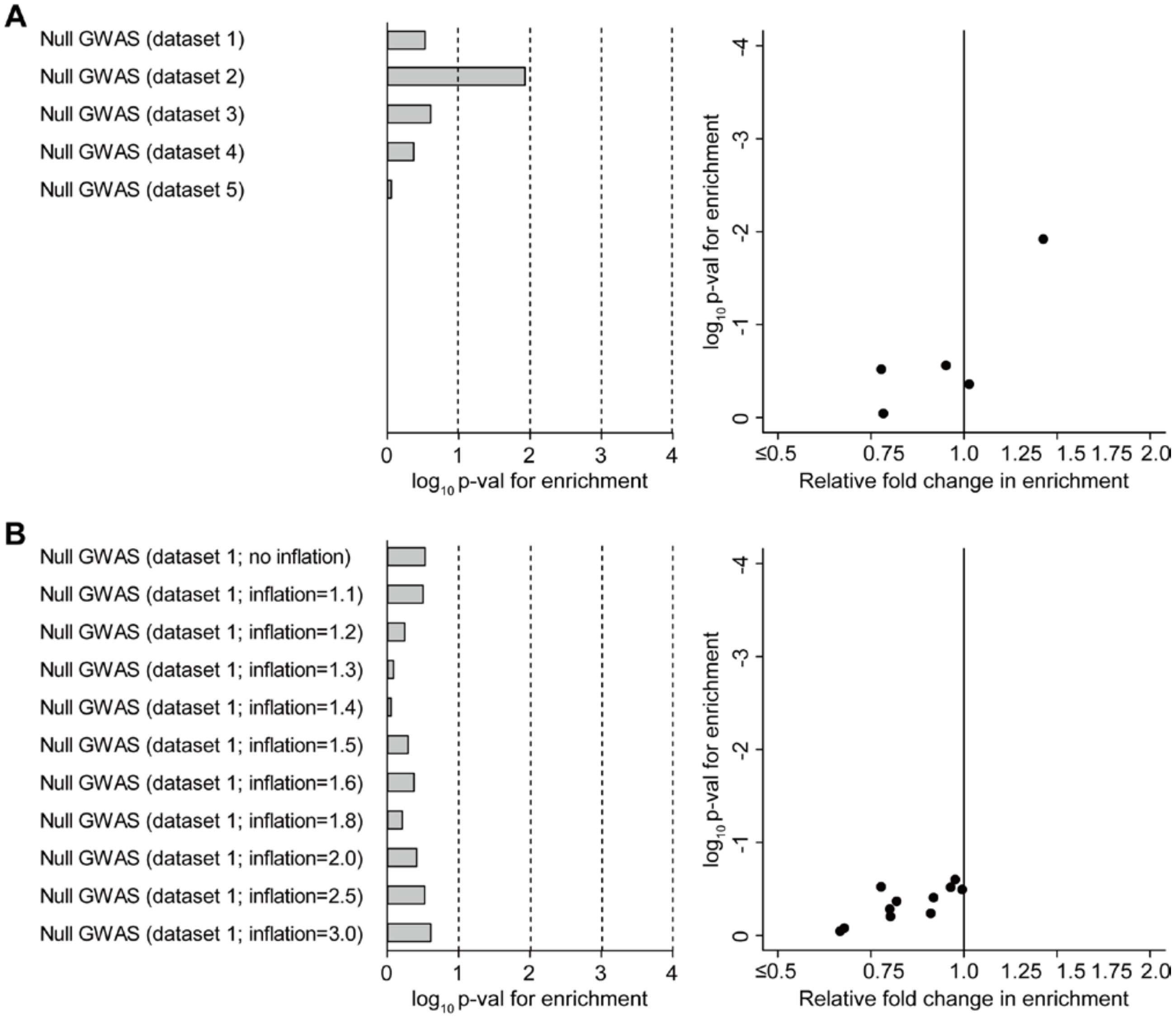

Significance (= $P_{enrichment}$) and relative fold changes (= $F_{enrichment}$) in the enrichment of the association signals of the null GWAS results on miRNA–target gene networks. (A) Results of data sets 1-5. (**B)** Results of data set 1 with inflation. No enrichment was observed even when we artificially induced inflation of the GWAS association signals, by inversely applying genomic control (GC) corrections with λ_{GC} values in the range of 1.0–3.0.

Supplementary Figure 2. The sequences of primers for constructing of each luciferase reporter plasmid of *PADI2*

Region 1	Forward	TTCTCGAGGTCCACTGTGGCACCAACGT
	Reverse	TTGTCGACAGGCACAGATACCCCAAATT
Region 2	Forward	TTCTCGAGTTCTGCTGACATGGACTGGA
	Reverse	TTGTCGACAATTCTTTGGGGAGCAGTTT
Region 3	Forward	TTCTCGAGAATCCTCTTGGCTTTCTCT
	Reverse	TTGTCGACATTGAACTCTCAGGTCACAAG
Region 4	Forward	TTCTCGAGATATCCATCTTCTCTTGCCT
	Reverse	TTGTCGACGTAGGCCAGGGTCCTTATGT
Region 5	Forward	TTCTCGAGCTCCTCTCCGGACATCAGAT
	Reverse	TTGTCGACTTCTGGCCCTAAAGGAACTT
Region 6	Forward	TTCTCGAGGTCTTCTTTAAACAGGCCCC
	Reverse	TTGTCGACACATGACAAACTCTAAGTCT
Region 7	Forword	TTCTCGAGTTAGTCTACATTAGGGGGAA
	Reverse	TTGTCGACTCTAGGCCTACATTTATAGA

Physical positions of the regions on *PADI2* are indicated in **Supplementary Figure 3**.

Supplementary Figure 3. MiR-4728-5p suppresses PADI2 protein expression through directly binding to the 3'-UTR region

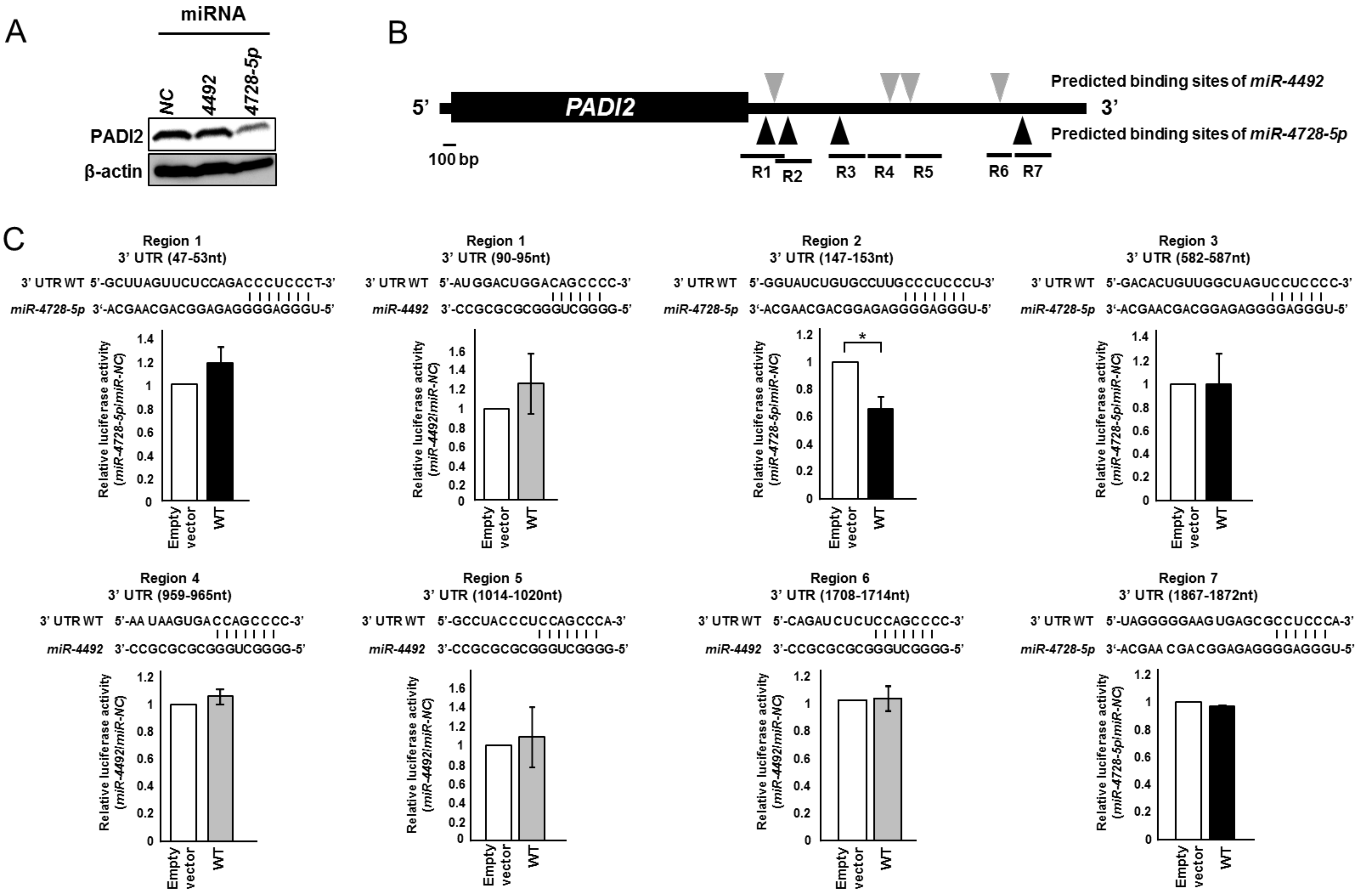

A: Protein expression level of PADI2 was confirmed by western blotting in MCF7 cells, were transfected each miRNA (negative control [NC], 4492, 4728-5p).

B: *PADI2* has eight putative binding sites of *miR-4492* and *-4728-5p* in its 3' UTR region. These sites were predicted by the miRNA target prediction databases.

C: Results of the luciferase reporter assays in HeLa cells after co-transfection with pmirGLO luciferase vectors containing each wild-type (WT) sequence and miR-4429 or 4728-5p. Experiments were performed in triplicate, and each data point represents the mean (bars, SD). Student's *t*-test was used for statistical analysis. An asterisk represents $P < 0.05$ versus each control transfectant.

Supplementary Figure 4. Cis-eQTL effect of rs761426 on *PADI2* mRNA expression levels

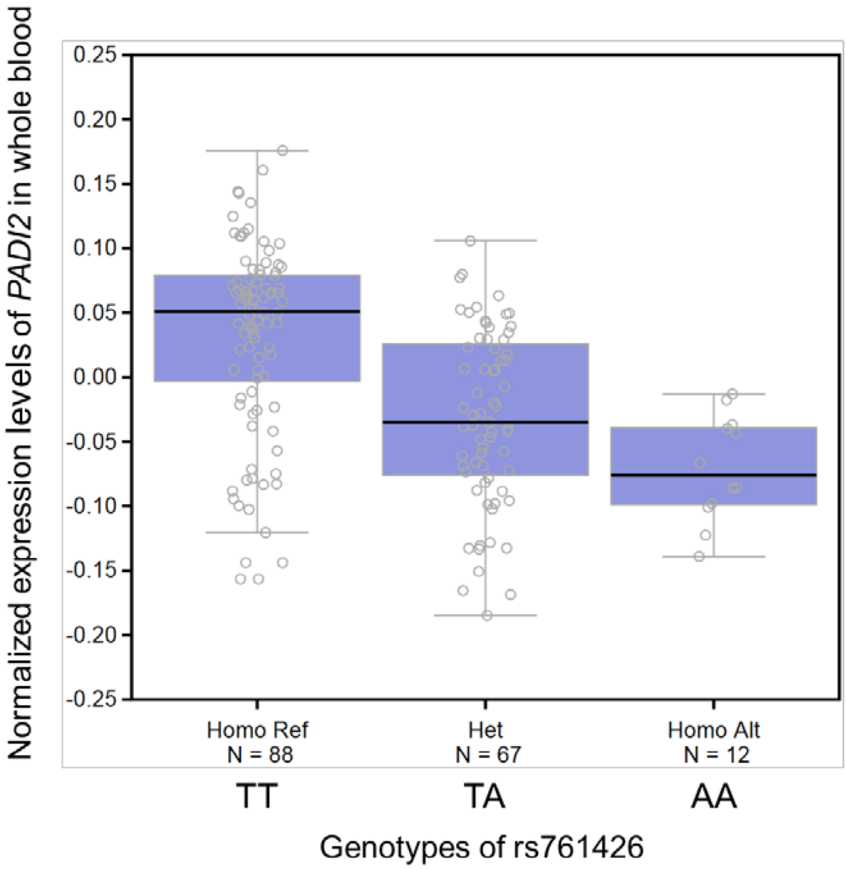

RA risk T allele of rs761426 significantly increases expression levels of *PADI2* in whole blood. Cis-eQTL analysis results of the SNP was obtained from Genotype-Tissue Expression (GTEx) Analysis Release v4 (dbGaP Accession phs000424.v4.p1).[20]

Supplementary Figure 5. The RA GWAS results at 17q12 that includes miR-4728-5p

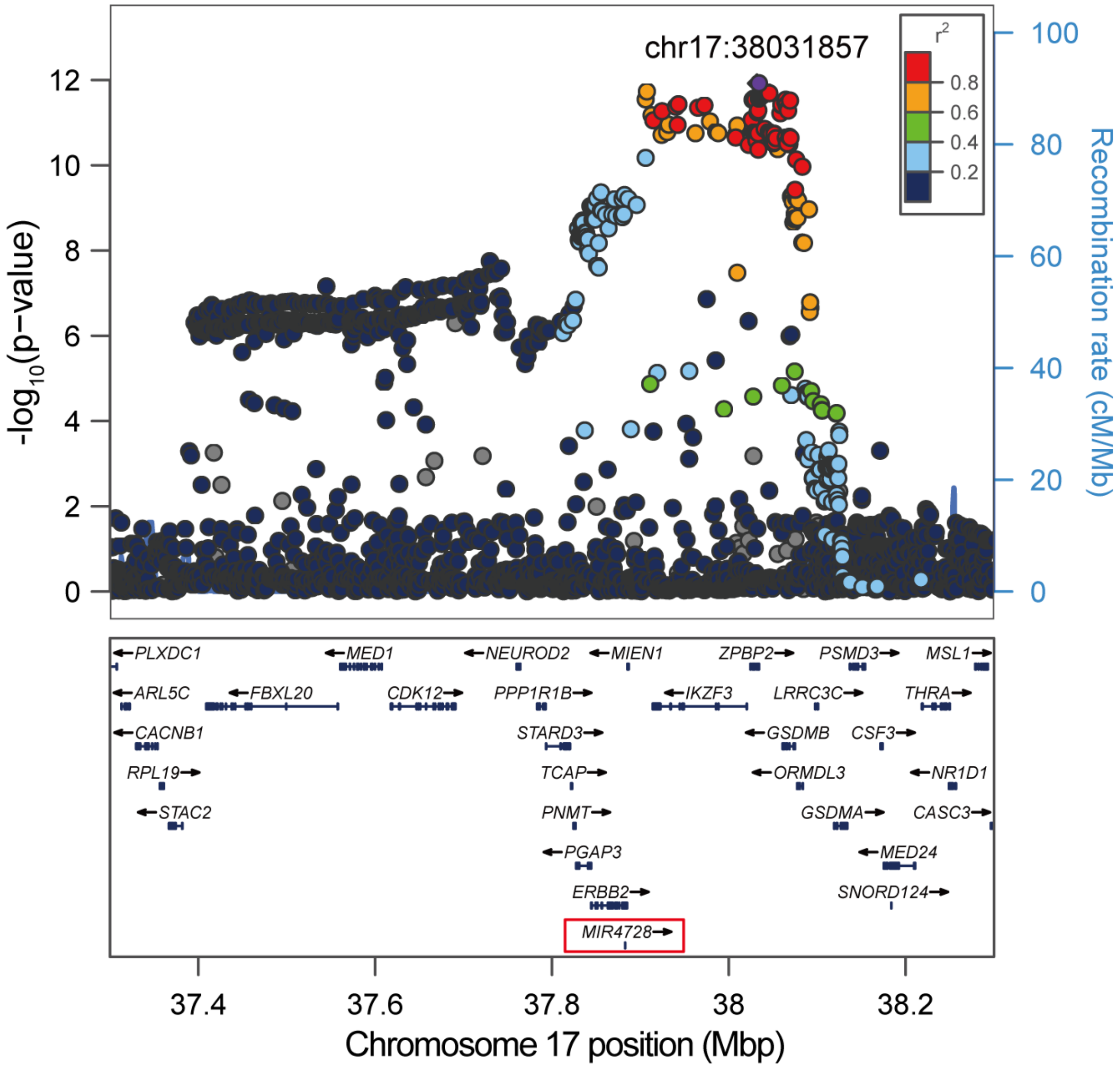

Each dot represents the $-\log_{10}$ P values of the SNPs at 17q12 obtained from the RA GWAS meta-analysis.[12] Color of the dot represents the r^2 value with the most significantly associated SNP as indicated in the legned. RefSeq genes are indicated below.

References

1. Wood AR et al. Defining the role of common variation in the genomic and biological architecture of adult human height. *Nat Genet* **46**, 1173-1186 (2014).
2. Perry JR et al. Parent-of-origin-specific allelic associations among 106 genomic loci for age at menarche. *Nature* **514**, 92-97 (2014).
3. Fritsche LG et al. Seven new loci associated with age-related macular degeneration. *Nat Genet* **45**, 433-9, 439e1-2 (2013).
4. Lambert JC et al. Meta-analysis of 74,046 individuals identifies 11 new susceptibility loci for Alzheimer's disease. *Nat Genet* **45**, 1452-1458 (2013).
5. Ehret GB et al. Genetic variants in novel pathways influence blood pressure and cardiovascular disease risk. *Nature* **478**, 103-109 (2011).
6. Locke AE et al. Genetic studies of body mass index yield new insights for obesity biology. *Nature* **518**, 197-206 (2015).
7. Estrada K et al. Genome-wide meta-analysis identifies 56 bone mineral density loci and reveals 14 loci associated with risk of fracture. *Nat Genet* **44**, 491-501 (2012).
8. Köttgen A et al. New loci associated with kidney function and chronic kidney disease. *Nat Genet* **42**, 376-384 (2010).
9. Willer CJ et al. Discovery and refinement of loci associated with lipid levels. *Nat Genet* **45**, 1274-1283 (2013).
10. Gieger C et al. New gene functions in megakaryopoiesis and platelet formation. *Nature* **480**, 201-208 (2011).
11. van der Harst P et al. Seventy-five genetic loci influencing the human red blood cell. *Nature* **492**, 369-375 (2012).
12. Okada Y et al. Genetics of rheumatoid arthritis contributes to biology and drug discovery. *Nature* **506**, 376-381 (2014).
13. Schizophrenia Working Group of the Psychiatric Genomics Consortium. Biological insights from 108 schizophrenia-associated genetic loci. *Nature* **511**, 421-427 (2014).
14. Mahajan A et al. Genome-wide trans-ancestry meta-analysis provides insight into the genetic architecture of type 2 diabetes susceptibility. *Nat Genet* **46**, 234-244 (2014).
15. Kottgen A et al. Genome-wide association analyses identify 18 new loci associated with serum urate concentrations. *Nat Genet* **45**, 145-154 (2013).
16. Wong N, Wang X. miRDB: an online resource for microRNA target prediction and functional annotations. *Nucleic Acids Res* **43**, D146-D152 (2015).
17. Vejnar CE, Zdobnov EM. MiRmap: comprehensive prediction of microRNA target repression strength. *Nucleic Acids Res* **40**, 11673-11683 (2012).
18. Kertesz M et al. The role of site accessibility in microRNA target recognition. *Nat Genet* **39**, 1278-1284 (2007).
19. Lewis BP, Burge CB, Bartel DP. Conserved seed pairing, often flanked by adenosines,

indicates that thousands of human genes are microRNA targets. *Cell* **120**, 15-20 (2005).

20. GTEx Consortium. Human genomics. The Genotype-Tissue Expression (GTEx) pilot analysis: multitissue gene regulation in humans. *Science* **348**, 648-660 (2015).
